# Evolving Paradigms in Cancer Pain Management: From Opioid-Centric Care to Multimodal and Personalized Strategies

**DOI:** 10.3390/cancers18091476

**Published:** 2026-05-03

**Authors:** Isabella Barrios, Sara A. Thomas, Yesenia L. Hernandez, Ana Pagan, Emily Munoz, Kamilah Cespedes, Saurabh Aggarwal

**Affiliations:** 1 Alabama College of Osteopathic Medicine, Dothan, AL 36303, USA; barriosi2894@acom.edu (I.B.); thomass9560@acom.edu (S.A.T.); 2 Department of Cellular and Molecular Medicine, Herbert Wertheim College of Medicine, Florida International University, Miami, FL 33174, USA; ylopezhe@fiu.edu (Y.L.H.); apaga039@fiu.edu (A.P.); emuno052@fiu.edu (E.M.); kcesp010@fiu.edu (K.C.)

**Keywords:** cancer pain, palliative care, multimodal analgesia, opioids, adjuvant analgesics, supportive oncology, personalized medicine

## Abstract

Background/Objectives: Cancer-related pain remains one of the most prevalent and distressing symptoms across the disease trajectory, significantly impairing function and quality of life. Although opioids are central to managing moderate to severe pain, their limitations, including adverse effects, dependence risk, and societal concerns, highlight the need for more individualized and comprehensive strategies. This review aims to synthesize current approaches to cancer pain management within a palliative care framework, emphasizing multimodal, mechanism-based, and patient-centered care. Methods: A narrative review was conducted using literature searches of PubMed, Scopus, and Google Scholar. Articles published between 2010 and 2026, with emphasis on recent literature (2020–2026), were included. Search terms included combinations of “cancer pain,” “palliative care,” “multimodal analgesia,” “opioids,” “adjuvant analgesics,” and “neuropathic pain.” Peer-reviewed studies, clinical guidelines, systematic reviews, and meta-analyses relevant to cancer pain mechanisms and management were considered. Results: Cancer pain is heterogeneous, arising from tumor progression and treatment-related injury, and includes neuropathic, visceral, and somatic components. Effective management requires mechanism-based assessment and multimodal strategies. Adjuvant analgesics, such as antidepressants, anticonvulsants, corticosteroids, and topical agents, enhance pain control and reduce opioid reliance. Non-pharmacological interventions and early integration of palliative care further improve symptom management and quality of life. Emerging therapies, including cannabinoid-based treatments and gene-targeted approaches, show promise but require further clinical validation. Conclusions: A multidisciplinary, patient-centered approach that integrates pharmacologic and non-pharmacologic strategies is essential for optimizing cancer pain management. Advancing toward personalized and multimodal care models may improve outcomes, reduce opioid-related risks, and enhance quality of life for patients with cancer.

## Simple Summary

Cancer pain is a common and often distressing problem that can greatly reduce a patient’s quality of life. Although strong pain medications like opioids are widely used, they can cause side effects and raise concerns about dependence. This review explores better ways to manage cancer-related pain by combining different types of treatments tailored to each patient’s needs. The authors aim to highlight the importance of using multiple approaches, including non-drug therapies, supportive care, and additional medications that enhance pain relief while reducing reliance on opioids. The review also discusses new and emerging treatments that may improve future care. By bringing together current knowledge and future directions, this work supports a more personalized and balanced approach to cancer pain management, which may guide clinicians and researchers toward safer and more effective strategies for improving patient well-being.

## 1. Introduction

With a steadily increasing global incidence, cancer remains a leading cause of mortality worldwide. Among its many associated challenges, chronic pain is one of the most prevalent and debilitating symptoms, significantly impairing quality of life and potentially affecting treatment adherence and outcomes. Despite advances in oncology and pain management, cancer-related pain remains complex and often inadequately controlled. An estimated 51.9% of individuals with advanced or metastatic cancer experience moderate to severe pain [1]. Cancer-related pain arises from heterogeneous mechanisms, including tumor progression and treatment-induced neuropathies. Many patients rely on opioids, raising concerns regarding adverse effects and dependence [2]. These challenges underscore the need for more individualized, mechanism-based approaches to pain management.

Over the past decade, several narrative reviews have examined cancer pain management, primarily focusing on pharmacologic treatment algorithms, including the use of opioids and adjuvant analgesics, as well as the role of non-pharmacological interventions and palliative care integration. While these reviews have contributed significantly to clinical practice, many continue to emphasize stepwise, opioid-centered frameworks or provide broad overviews without fully integrating emerging concepts such as mechanism-based classification, multimodal care models, and precision medicine approaches. This review builds upon prior literature by offering a more comprehensive and integrative perspective on cancer pain management. Specifically, we (1) incorporate a mechanism-based classification of pain, including neuropathic, visceral, and somatic subtypes to guide targeted therapy; (2) emphasize multimodal and opioid-sparing strategies, combining pharmacologic, non-pharmacologic, and interventional approaches; and (3) contextualize pain management within broader challenges, including health disparities, the evolving opioid landscape, and advances in precision medicine, such as pharmacogenomics and gene-targeted therapies. By bridging established clinical approaches with emerging evidence and future directions, this narrative review provides a forward-looking framework to optimize pain control, improve functional outcomes, and enhance quality of life in patients with cancer.

## 2. Methods

**Figure 1:**
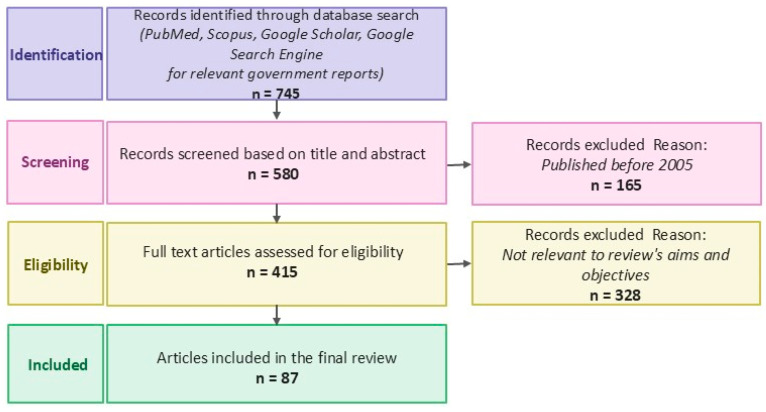
PRISMA flow chart.

This manuscript was conducted as a narrative review to synthesize current evidence on cancer pain mechanisms and management strategies within a palliative care framework. A comprehensive literature search was performed using the following electronic databases: PubMed, Scopus, and Google Scholar. The search was conducted to identify relevant peer-reviewed articles published between January 2010 and March 2026, with an emphasis on more recent studies (2020–2026) to reflect current clinical practice and evolving evidence. Search terms were developed to capture key domains of cancer pain and its management. Keywords and combinations included: cancer pain, oncologic pain management, palliative care, multimodal analgesia, opioids, adjuvant analgesics, neuropathic pain, visceral pain, somatic pain, cannabinoids, pharmacogenomics, gene-targeted therapy. Boolean operators (AND, OR) were used to refine searches and combine concepts. Articles were selected based on relevance to the topic and clinical applicability. The inclusion criteria were: (1) Peer-reviewed journal articles, systematic reviews, meta-analyses, and clinical guidelines, (2) Studies addressing mechanisms, assessment, or management of cancer-related pain, (3) Publications in English, (4) Studies involving adult cancer populations, and (5) Relevant guidelines from organizations such as the American Society of Clinical Oncology and European Society for Medical Oncology. The Exclusion criteria were: (1) non-English publications, (2) Case reports with limited generalizability, (3) Studies not directly related to cancer pain or its management, and (4) Articles with insufficient methodological detail or unclear relevance (Figure 1). Articles were screened based on title and abstract, followed by full-text review for relevance. Given the narrative nature of this review, formal quality scoring and meta-analysis were not performed. Instead, studies were selected to provide a balanced and comprehensive overview of established practices, emerging therapies, and ongoing controversies in cancer pain management.

## 3. Common Cancers Associated with Pain

Cancer progression is frequently associated with the development of chronic pain, though its prevalence and characteristics vary by malignancy. Rather than being limited to specific cancer types, pain is a common feature across many solid tumors, as well as hematologic malignancies, and arises from a combination of tumor burden, metastatic spread, and treatment-related effects. Among solid tumors, malignancies such as lung, breast, prostate, and gastrointestinal cancers are particularly associated with a high pain burden due to their propensity for local invasion, nerve involvement, and bone metastases [3]. Understanding both shared and tumor-specific pain mechanisms is essential for guiding effective, mechanism-based management strategies.

### 3.1. Solid Tumors and Pain Mechanisms

Pain in solid tumors commonly results from tumor infiltration into surrounding tissues, including bone, nerves, and visceral organs. Thoracic malignancies, such as lung cancer, may produce pain through chest wall invasion, pleural irritation, or compression of neural structures, sometimes leading to syndromes such as intercostal neuralgia or brachial plexopathy. Similarly, abdominal and pelvic tumors may involve lumbosacral plexus, resulting in neuropathic pain syndromes. Bone metastases, frequently observed in cancers such as breast and prostate cancer, are a major contributor to somatic pain and are often associated with significant morbidity.

### 3.2. Lung and Breast Cancer as Representative Models

Lung and breast cancers remain among the most prevalent malignancies worldwide and are frequently used as clinical models for studying cancer-related pain due to their high symptom burden and diverse pain mechanisms [4]. Individuals with advanced-stage lung cancer often necessitate palliative care due to metastatic spread or progression of the primary tumor. Despite the utilization of diverse therapeutic modalities, opioid administration remains prevalent due to the inherent complexities of adequate pain control. In a study of 152 lung cancer patients, 73.7% of those at stage IV experienced moderate (72.4%) or severe pain (27.6%), with chest or back pain reported in 76.3% of cases and neuropathic pain noted in 46.7% of patients [5]. Cancer-related pain in lung cancer manifests as both acute pain and chronic pain syndromes, arising from several mechanisms. These include tumor infiltration into the chest wall and pleura, nerve compression resulting from tumor impingement on structures such as the brachial plexus, and skeletal pain secondary to bone metastases.

Although lung cancer is frequently linked to elevated incidences of chronic pain, patients with breast cancer also experience substantial pain attributable to malignancy and its associated therapeutic interventions. Multiple studies have investigated the prevalence and intensity of pain experienced by breast cancer survivors. For instance, a survey conducted by Hamood et al. with 305 breast cancer survivors found that 84% of participants reported experiencing moderate pain, while 97% experienced pain on one to three days per week or more. Furthermore, a significant proportion of patients reported pain-associated symptoms such as paresthesia, allodynia, and phantom sensations, underscoring the persistent and multifaceted nature of pain in breast cancer survivors [6]. Pain mechanisms in breast cancer include tumor infiltration into the chest wall, nerves, and lymph nodes, which can cause localized and neuropathic pain. Post-mastectomy pain syndrome, a common consequence of breast surgery, is characterized by chronic, burning pain localized to the chest or axillary region, attributed to nerve injury [7]. Additionally, bone metastases frequently contribute to skeletal pain, particularly in the spine, ribs, and pelvis, further complicating pain management in these patients [8].

### 3.3. Hematologic Malignancies

Pain has long been a neglected aspect in malignant hematology [9,10]. Most patients with hematologic malignancies experience a significant symptom burden, frequently including severe pain, typically present at disease onset. As *Niscola* et al. explained in their study, some patients report pain relief following the initiation of chemotherapeutic agents; however, survivors or those in advanced disease stages commonly complain of chronic pain due to treatment-related complications and the disease itself [11]. In patients with leukemia, bone pain may result from the expansion of bone marrow caused by the accumulation of abnormal white blood cells. Depending on the affected site, it can manifest as either sharp or dull discomfort [12]. The long bones of the legs and arms are common sites of this pain. Conversely, lymphoma typically presents as painless lymphadenopathy. In advanced stages, B symptoms, such as unexplained fever, significant weight loss, and drenching night sweats, may occur, mediated by pro-inflammatory cytokines [13]. Patients experience diverse pain types, including neuropathic, visceral, and somatic pain, which are crucial for effective management.

## 4. Pain Presentations

Cancer-related pain is a heterogeneous and multifactorial experience that spans both solid tumors and hematologic malignancies. It is broadly classified into neuropathic, visceral, and somatic pain, although these categories frequently overlap within the same patient. Rather than being defined solely by cancer type, pain presentations are more accurately understood through their underlying mechanisms, which may include tumor invasion, metastatic spread, inflammation, and treatment-related injury (Table 1). Recognizing these patterns is essential for guiding mechanism-based and multimodal pain management strategies.

### 4.1. Neuropathic Pain

Neuropathic pain results from injury or dysfunction of the somatosensory system and is commonly encountered across a wide range of malignancies [14]. It may arise in both solid tumors and hematologic cancers due to direct tumor invasion, compression of neural structures, or treatment-related neurotoxicity. Clinically, patients often present with burning, shooting, or electric shock-like sensations, frequently accompanied by allodynia, paresthesia, and sensory deficits [15]. Neuropathic pain can be either central or peripheral, depending on the origin of the neural lesion. Central neuropathic pain originates from disorders within the central nervous system, including the brain and spinal cord, in conditions like stroke and Parkinson’s disease [16]. Conversely, the peripheral neuropathic pain predominantly involves abnormalities in peripheral afferent fibers, specifically small, unmyelinated C-fibers and myelinated A-fibers (Aβ and Aδ) [17]. Peripheral neuropathic pain is expected to become more prevalent due to the aging global population, rising cancer incidence, and the effects of chemotherapy, which impact various sensory nerve fibers (Aβ, Aδ, and C fibers) [18].

In cancer patients, the etiology of neuropathic pain is multifactorial: approximately two-thirds are directly tumor-related, 20% are treatment-induced, and 10–15% are attributable to comorbidities [19]. Neuropathic cancer pain can arise from direct infiltration of the peripheral or central nervous system by primary tumors or metastases. Examples of peripheral nervous system involvement include brachial plexus invasion by thoracic tumors and lumbosacral plexus invasion by abdominal or pelvic malignancies [20]. Chemotherapy-induced peripheral neuropathy (CIPN) demonstrates significant variability in both incidence and severity depending on the type of malignancy and the specific chemotherapeutic agents used. Cancers commonly treated with neurotoxic agents, such as lung, breast, colorectal, and hematologic malignancies, are associated with a higher risk of CIPN. In particular, regimens containing platinum compounds (e.g., cisplatin, oxaliplatin), taxanes (e.g., paclitaxel, docetaxel), and vinca alkaloids (e.g., vincristine) are strongly linked to the development of neuropathy. The risk is further influenced by cumulative dose, treatment duration, and patient-specific factors such as age and comorbidities. Notably, CIPN may persist long after treatment cessation, with a substantial proportion of patients experiencing chronic neuropathic symptoms that significantly impact functional status and quality of life. A recent meta-analysis of 76 studies from 29 countries with 13,635 patients with CIPN showed that the pooled prevalence of those reporting chronic moderate-to-severe CIPN or painful CIPN was estimated at 47.76% [21]. CIPN often presents as symmetric distal sensory loss, tingling, or pain, significantly impacting long-term quality of life [22]. Radiation-induced neuropathy represents another significant pathway to chronic neuropathic pain, although its precise mechanisms remain incompletely elucidated. Proposed etiologies include nerve compression secondary to radiation-induced fibrosis and direct neural and microvascular injury resulting from alterations in the vasculature [23]. Radiation-induced neuropathy is classically exemplified by brachial plexopathy that develops after thoracic irradiation, often for lung or breast cancer [24].

### 4.2. Visceral Pain

Visceral pain arises when internal organs within the gastrointestinal or genitourinary systems are affected, including hollow organs, solid organs, the peritoneum, or tissues in the retroperitoneal space. It is commonly triggered by blockages in structures like the intestines, bile ducts, or ureters, leading to often diffuse pain. In women, reproductive physiology contributes to the addition of visceral pain throughout life [25]. Visceral pain in cancer presents with characteristics distinct from neuropathic pain. Tumor growth can generate multiple nociceptive stimuli that contribute to pain. These include the release of chemical substances by cancer and immune cells, physical pressure from tumor growth, blockages in hollow organs, and nerve-related changes such as loss of nerve supply, abnormal nerve growth, and altered nerve activity [25]. In hematologic malignancies, visceral compression due to enlarged lymph nodes and organomegaly, most commonly involving the spleen and/or liver, can lead to increased intra-abdominal pressure and associated discomfort [11].

### 4.3. Somatic Pain

Somatic pain is the most common type of pain observed in cancer patients, presenting as a dull, gnawing, pounding, or gripping discomfort [26]. Cutaneous somatic pain is typically sharp or burning and well-localized from lacerations. Conversely, deep somatic pain originates from joints, tendons, and bone metastasis, tends to be more diffuse, and is commonly characterized as throbbing or aching [25,27,28,29,30]. Somatic pain is the dominant pain subtype for lung and breast cancers due to bone metastasis. Hematologic malignancies present unique pain profiles due to bone marrow infiltration, skeletal involvement, and systemic symptoms. Among patients with hematological cancers, bone pain is one of the most significant forms of disease-related pain. It primarily arises from two key pathological mechanisms: the formation of osteolytic lesions and the infiltration of malignant cells into the bone marrow [31]. These bone lesions can cause constant nociceptive pain that may be localized or radiate to other areas, even during rest. The pain is often intensified by movement (incident pain) and may also include neuropathic characteristics, resulting in a mixed pain profile [11]. Currently, a hematologist in clinical practice can anticipate that chronic pain will be mentioned in approximately one out of every five patients [32]. Among patients undergoing active treatment, ongoing pain is observed in approximately 60%, and even during survivorship, persistent pain affects roughly 33% of individuals [33]. Importantly, pain in these patients may also be treatment-related, emerging postoperatively or as a side effect of chemotherapy or radiation therapy. Considering the substantial burden of chronic pain in cancer patients, particularly those with lung, breast, and hematological cancers, implementing effective pain management strategies is vital in optimizing clinical results and enhancing overall well-being.

## 5. Pain Therapies: A Mechanism-Based Approach

Effective cancer pain management requires a mechanism-based, multimodal strategy that aligns treatment selection with the underlying type of pain: neuropathic, visceral, somatic, or mixed. Given the heterogeneity of cancer-related pain, reliance on a single modality is often insufficient. Instead, combining pharmacological, non-pharmacological, and interventional approaches allows for improved analgesia while minimizing adverse effects, particularly opioid-related toxicity.

### 5.1. Non-Pharmacological Approaches

Non-pharmacological interventions play a critical role in addressing both the physical and psychosocial dimensions of cancer pain and are applicable across all pain types. These strategies are particularly valuable in patients with chronic or mixed pain syndromes, where psychological and behavioral factors significantly influence pain perception. Cognitive behavioral therapy (CBT) is the most utilized psychological approach for managing chronic pain. This therapy emphasizes the interplay between psychological state, behaviors, and pain awareness. Patients are guided to explore their emotional responses to pain, develop methods for managing stress and calming practices, and build decision-making skills, and to practice these regularly. CBT is especially beneficial in patients with chronic neuropathic and mixed pain, where central sensitization and maladaptive pain responses are common [34]. CBT specifically targets maladaptive beliefs surrounding pain and cancer, often leading to improved pain tolerance and emotional resilience [35]. CBT that incorporates imagery and hypnosis has shown the greatest potential to date in managing chronic pain associated with cancer. Patients are taught to use guided imagery to focus on a relaxing scene, diverting attention from pain. This intervention has displayed significant effectiveness in pediatric patients undergoing invasive procedures, including lumbar punctures and bone marrow aspirations [36], and has also been shown to aid in managing discomfort among females with advanced-stage breast cancer and individuals receiving stem cell transplants [37]. A randomized controlled trial (RCT) further supports the utility of CBT, imagery, and relaxation techniques in mitigating chemotherapy-associated side effects and pain [38].

Other modalities, including transcutaneous electrical nerve stimulation (TENS), physical therapy, massage, and thermal therapies (heat and cold), may provide adjunctive relief. TENS is thought to modulate nociceptive signaling and may be beneficial in somatic and neuropathic pain conditions. Acupuncture has also been explored as an adjunctive therapy, with some evidence suggesting modest benefits in chronic pain conditions. A meta-analysis evaluating acupuncture’s efficacy for various chronic pain conditions demonstrated statistically significant pain relief compared to both sham and no-acupuncture controls [39]. These findings propose that acupuncture could function as a valuable adjunctive therapy to conventional analgesics, potentially contributing to enhanced symptom control and improved patient well-being for cancer patients.

### 5.2. Pharmacological Approaches

Pharmacologic therapy remains the cornerstone of cancer pain management but is most effective when tailored to the underlying pain mechanism. This method advocates for the judicious use of nonopioid, opioid, and adjuvant analgesics, which can be administered as monotherapy or in combination. Dosing regimens are carefully titrated to each patient’s individual needs to optimize efficacy while minimizing adverse effects [40]. Opioids remain essential for moderate to severe cancer pain, particularly in visceral and somatic pain syndromes associated with tumor burden or metastases. Agents such as morphine, oxycodone, fentanyl, methadone, and hydromorphone are widely used due to their potent analgesic effects [41,42,43]. However, opioids are generally less effective as monotherapy for neuropathic pain, where additional agents are often required. Their use is limited by adverse effects, including gastrointestinal, neurological, and respiratory complications [44]. Long-term opioid therapy is associated with risks such as dependence, overdose, and opioid-induced hyperalgesia, necessitating careful patient selection and monitoring. These risks are dose-dependent, with higher doses associated with increased morbidity [45]. Despite these concerns, opioids remain appropriate, particularly in advanced disease, where the primary goal is symptom relief and quality of life. In this context, the focus shifts from long-term risk mitigation to effective pain control and patient comfort.

Non-Opioid Analgesics such as nonsteroidal anti-inflammatory drugs (NSAIDs) and acetaminophen are also particularly useful in somatic pain, especially pain arising from inflammation or bone metastases. NSAIDs may also provide benefit in visceral pain associated with inflammatory processes. These agents are often used in combination with opioids to enhance analgesia and reduce opioid requirements.

## 6. Adjuvant Analgesics and Multimodal Strategies in Cancer Pain Management

**Figure 2:**
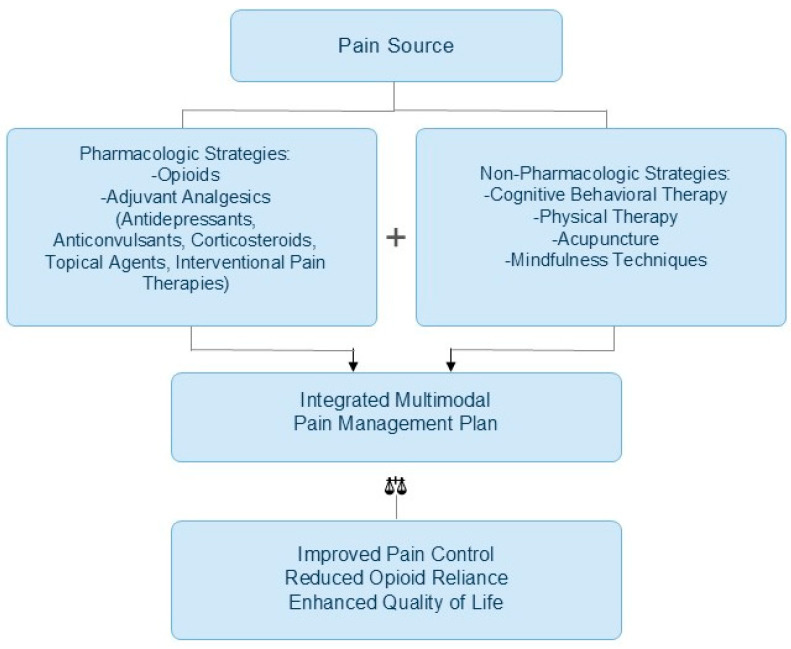
Multimodal approach to chronic cancer pain management. Pharmacologic and non-pharmacologic strategies converge into an integrated plan, aiming to improve pain control, reduce opioid reliance, and enhance quality of life.

In the last couple of years, the incorporation of adjuvant analgesics and multimodal techniques has emerged as a potential approach to enhance pain control, reduce opioid dependency, and tailor treatment to neuropathic, bone, or inflammatory pain syndromes. (Figure 2).

In the context of cancer, where pain often arises from multifactorial causes, targeted pharmacologic approaches are crucial in managing diverse pain syndromes. Adjuvant analgesics are medications that were not initially developed to treat pain, but they have been shown to possess pain-relieving properties in certain conditions [46]. When administered alone, these agents typically exhibit little or no analgesic activity but can significantly enhance their effects when combined with conventional analgesics [47]. These medications are generally used when pain does not respond to standard treatments. However, in particular chronic pain conditions, they may be considered as an initial treatment option [48]. The role of adjuvant analgesics is multifaceted. They can increase the therapeutic index of opioids by enabling lower dosing, providing analgesic effects where opioids are insufficient, and mitigating some opioid-related side effects. Due to these benefits, they are often an important part of personalized pain management, especially in patients who cannot tolerate high doses of opioids or in whom opioids are not the best option [49]. The World Health Organization’s (WHO) guidelines on cancer pain management recommend the use of adjuvant medicines, including steroids, antidepressants, and anticonvulsants, to heighten the impact of opioids and NSAIDs and to manage specific pain syndromes [50]. These adjuvant therapeutic analgesics can alleviate bone-related, inflammatory-related, and neuropathic pain states.

### 6.1. Anticonvulsants

Neuropathic pain, regardless of the origin, is best described due to increased nerve activity in injured neural circuits. This hyperexcitability and the associated molecular changes closely resemble those observed in particular types of epilepsy, which has prompted the repurposing of anticonvulsant medications for neuropathic pain management [51]. Drugs such as gabapentin and pregabalin are commonly prescribed to manage neuropathic pain, especially when it results from nerve compression or side effects of chemotherapy [52]. Gabapentin is particularly effective in relieving neuropathic pain, especially in cases such as painful diabetic neuropathy and postherpetic neuralgia. Given its proven efficacy and favorable side effect profile, it is often regarded as a first-line treatment option for managing neuropathic pain [53]. The gabapentinoids, initially developed as analogs of gamma-aminobutyric acid (GABA), bind to the α2δ-1 and α2δ-2 auxiliary subunits of calcium channels [53], therefore leading to a decrease in the release of stimulating neurotransmitters, and helps calm overactive nerve activity. These drugs are usually well tolerated, with dizziness and drowsiness being the most frequently reported side effects.

### 6.2. Antidepressants

Tricyclic antidepressants (TCAs), such as imipramine, amitriptyline, and clomipramine, are especially effective in neuropathic pain due to their dual inhibition of serotonin and norepinephrine reuptake. Their serotonin reuptake inhibition is primarily due to the parent compounds, while the norepinephrine reuptake effects are largely mediated by their active metabolites, nortriptyline, desipramine, and desmethylclomipramine. Notably, nortriptyline and desipramine are also used independently as TCAs, and along with maprotiline, they primarily target norepinephrine reuptake [54]. Evidence supports the efficacy of TCAs in treating a variety of peripheral pain syndromes involving nerve damage, including painful diabetic and non-diabetic polyneuropathies, postherpetic neuralgia, and postmastectomy pain syndrome [54]. Additionally, serotonin–norepinephrine reuptake inhibitors (SNRI) like duloxetine are commonly used for chemotherapy-induced peripheral neuropathy and are often preferred because they tend to cause fewer or milder side effects than tricyclic antidepressants [55]. Given their well-established efficacy in treating various neuropathic pain syndromes, both TCA and SNRIs remain valuable components of cancer pain management, particularly in patients experiencing nerve damage from chemotherapy, surgery, or tumor infiltration.

### 6.3. Corticosteroids

Corticosteroids are frequently prescribed in palliative care settings for individuals with cancer [56]. In a Canadian study involving outpatient palliative care cancer patients, 40% were found to be using corticosteroids, with dexamethasone being the most frequently prescribed by palliative care providers [57]. By interfering with key biochemical and cellular pathways involved in inflammation and immune responses, corticosteroids (CS) exert anti-inflammatory, anticancer, and immunosuppressive effects. One of their actions includes increasing annexin A1 levels, which leads to suppression of phospholipase A2 (PLA2) activity and a reduction in prostaglandin and leukotriene production. They also help control inflammation by lowering cyclooxygenase-2 (COX-2) expression and restricting neutrophil movement to inflamed tissues [58]. The most common types of corticosteroids are dexamethasone and prednisone, which are broadly used in cancer pain for their anti-inflammatory and edema-reducing effects. Corticosteroids help control pain resulting from tumor-induced inflammation due to their anti-inflammatory action. They are also utilized in treating cancer-related conditions such as spinal cord compression and metastases to the brain [59]. Corticosteroids have other beneficial effects, such as increased appetite, general well-being, and social interaction [60]. While effective for systemic inflammation and deep tissue pain, corticosteroids may be less suitable for localized pain, such as that affecting the skin or peripheral nerves, where topical agents can offer more targeted relief with fewer systemic effects.

### 6.4. Topical Agents

Topical analgesics offer a localized approach to pain management, which is particularly useful for patients experiencing focal neuropathic pain where systemic medication may not be necessary or tolerated. Survey data indicate that over a quarter of physicians utilize compounded topical formulations as pain management strategies. Notably, approximately 43% of patients demonstrated favorable outcomes with these treatments, which were associated with a low incidence of adverse effects [61]. When applied to the skin, topical agents can achieve adequate drug levels in the targeted tissues while minimizing systemic absorption, resulting in relatively low plasma concentrations [62]. Keratinocytes, making up approximately 90% of the epidermal cell population, represent a primary target for topical pain-relieving agents. Although typically classified as non-excitable cells, keratinocytes play a significant role in mediating cutaneous responses. Keratinocytes play a key role by releasing substances that activate pain nerves when injured. Injury to peripheral tissues stimulates keratinocytes and dermal blood vessels to release excitatory mediators, such as substance P, calcitonin gene-related peptide (CGRP), and prostaglandins, which interact with receptors on nociceptive neurons, leading to their depolarization [63].

Lidocaine acts as a non-selective blocker of voltage-gated sodium channels, interfering with the initiation and transmission of nerve signals. It stabilizes neuronal membranes, helping suppress abnormal activity in damaged sensory neurons. Its analgesic effects may also involve modulation of keratinocytes and immune cells and interaction with irritant receptors such as transient receptor potential vanilloid 1 (TRPV1) and transient receptor potential ankyrin 1 (TRPA1) [64]. In addition to lidocaine patches, capsaicin-based treatments are also utilized for managing localized pain. Low-concentration capsaicin creams, which require regular daily application, have demonstrated modest yet reproducible analgesic benefits, as supported by multiple meta-analyses. More recently, a high-concentration capsaicin 8% patch, Qutenza™, has gained approval in both the United States and the European Union. This formulation offers several advantages, including a longer-lasting analgesic effect, improved patient adherence, and a low likelihood of causing widespread side effects or interacting with other medications. The analgesic mechanism of capsaicin is primarily attributed to the depletion of substance P, a neuropeptide integral to the transmission of nociceptive signals, within primary afferent neurons [65]. In cancer patients, topical agents may be particularly valuable for managing focal pain syndromes such as post-surgical neuropathy or chemotherapy-induced dermal pain, offering relief without the burden of systemic side effects.

### 6.5. Interventional Pain Management

For patients with refractory cancer pain that does not respond adequately to pharmacological and non-pharmacological therapies, interventional approaches are an important component of multimodal care. These include: (1) Peripheral nerve blocks and steroid injections for localized somatic or neuropathic pain, (2) Neurolytic procedures (e.g., celiac plexus block) for severe visceral pain, (3) Intrathecal drug delivery systems, allowing direct administration of analgesics to the central nervous system, and (4) Spinal cord stimulation and peripheral nerve stimulation, which modulate pain signaling pathways. Interventional therapies are particularly valuable in patients with well-localized pain syndromes or opioid-refractory pain, offering significant analgesia while reducing systemic medication burden [66].

## 7. Multimodal Pain Management

Multimodal pain management integrates therapies targeting different biological pathways to enhance analgesic efficacy. Combining pharmacologic agents, such as opioids, with antidepressants or anticonvulsants, with non-pharmacological interventions can produce synergistic effects while reducing opioid requirements [67]. Examples of combination therapies include the following: for neuropathic + somatic pain, opioid + anticonvulsant + NSAID; for visceral + inflammatory pain, opioid + corticosteroid; and for focal neuropathic pain, systemic therapy + topical agent or nerve block. This strategy has significantly improved contemporary cancer pain management by enhancing analgesia while reducing opioid requirements.

In addition to pharmacologic strategies, non-pharmacologic interventions, such as CBT and various other techniques, are integral to multimodal pain management. Non-invasive, non-drug interventions (NINPT) provide effective and safe methods for managing chronic pain, minimizing tissue damage, and reducing the risk of major side effects [68]. Techniques such as transcutaneous electrical nerve stimulation (TENS), thermal therapies including heat and cold applications, and massage are designed to influence chronic pain signaling by engaging underlying physiological processes [69,70]. Interventions grounded in psychological frameworks, including mindfulness-based stress reduction and CBT, have demonstrated effectiveness in reducing pain perception and improving coping mechanisms in patients with chronic conditions [38,71]. Collectively, these multimodal strategies not only address physical pain but also help manage the emotional and psychosocial dimensions of suffering. The multimodal approach allows for a customized approach that aligns with the individual’s specific pain patterns and the nature of their illness.

### 7.1. Palliative Care and Interdisciplinary Approaches to Cancer Pain

An essential yet sometimes underutilized component of multimodal cancer pain management is the early integration of palliative care. Palliative care aims to enhance a patient’s overall well-being by alleviating distressing symptoms, providing psychosocial support, and facilitating goal-oriented communication. Pain is often the most distressing symptom managed by palliative teams, and their involvement has been associated with improved symptom control, reduced hospital admissions, and even increased survival in some cases [72,73]. The interdisciplinary nature of palliative care teams, typically comprising physicians, nurses, psychologists, social workers, chaplains, and pharmacists, allows for a multidimensional approach to pain management. This team-based structure is particularly beneficial in addressing the complex interaction between emotional, physical, spiritual, and societal aspects of cancer-related pain [74].

Despite these benefits, significant gaps remain in the consistent and timely delivery of palliative care services. The American Society of Clinical Oncology (ASCO) has released guidelines that underscore the value of a multidisciplinary palliative care approach in advanced cancer, highlighting its contributions to effective pain management, enhanced quality of life, and comprehensive symptom control [75]. Moreover, hospice services, which fall under the broader umbrella of palliative care, play a critical role in managing refractory pain at the end of life. Hospice services frequently employ individualized pain management plans and continuous infusions to ensure optimal comfort during the final stages of illness [76]. Incorporating palliative care into cancer pain management enhances patient-centered outcomes and exemplifies a shift toward a holistic, multidisciplinary approach that prioritizes both longevity and quality of life.

### 7.2. Real-World Limitations of Multimodal Cancer Pain Management

Although multimodal pain management is widely advocated as the optimal approach for cancer-related pain, its implementation in routine clinical practice is frequently constrained by several real-world barriers that limit its consistency, accessibility, and effectiveness. A primary limitation is unequal access to interdisciplinary care. Effective multimodal management relies on coordinated input from oncology, palliative care, pain specialists, psychology, rehabilitation services, and nursing. However, such integrated teams are not universally available, particularly in low-resource settings, rural regions, and non-tertiary care centers. Even in high-income healthcare systems, delays in referral to palliative care or pain specialists often result in fragmented care, where pharmacological management dominates while psychological and rehabilitative interventions remain underutilized.

Cost and resource constraints further limit implementation. Non-pharmacological interventions such as CBT, physical therapy, acupuncture, and interventional pain procedures require trained personnel, infrastructure, and sustained funding. These services may not be covered fully by insurance systems, leading to disparities in uptake. Similarly, advanced interventions such as intrathecal drug delivery systems or neuromodulation techniques may be restricted to specialized centers due to high procedural and maintenance costs, limiting their broader applicability.

A third major challenge is variability in provider expertise and clinical practice patterns. Multimodal pain management requires familiarity with multiple pharmacologic classes, interventional techniques, and psychosocial approaches. However, training in pain medicine and palliative care remains inconsistent across institutions. As a result, clinicians may preferentially rely on opioid-based strategies rather than fully integrating adjuvant analgesics or non-pharmacological modalities. This variability contributes to heterogeneity in care quality and may partially explain the persistent undertreatment of cancer pain despite established guidelines.

In addition, fragmented healthcare systems and limited communication between specialties can hinder coordinated decision-making, reducing the effectiveness of multimodal strategies even when individual components are available. Collectively, these limitations highlight a gap between guideline-recommended multimodal cancer pain management and its real-world implementation. Addressing these challenges will require expanded access to palliative and pain services, improved reimbursement structures for non-pharmacological therapies, and enhanced interdisciplinary training to ensure that multimodal pain management can be delivered consistently and equitably across diverse clinical settings.

## 8. Controversies and Evolving Paradigms in Cancer Pain Management

Cancer pain management continues to evolve within a complex clinical and sociopolitical context, where established therapeutic approaches are increasingly shaped by concerns surrounding opioid safety, health equity, and the integration of emerging therapies. While opioids, non-pharmacological strategies, and novel agents remain central to care, significant controversies persist regarding their optimal use and implementation.

### 8.1. Opioid Prescribing, Safety Concerns, and Stigma

Opioids remain the cornerstone of treatment for moderate to severe cancer-related pain; however, their use is increasingly influenced by concerns derived from the broader opioid crisis. Although cancer pain is a distinct clinical entity from chronic non-cancer pain, opioid prescribing patterns have been affected by heightened regulatory scrutiny, restrictive prescribing policies, and clinician hesitancy. Evidence suggests that these factors may contribute to undertreatment of cancer pain, particularly in outpatient and community settings. Some studies report delays in opioid initiation and subtherapeutic dosing due to fear of regulatory consequences or patient misuse, despite guideline recommendations supporting their use in advanced cancer. At the same time, legitimate concerns persist regarding opioid-related adverse effects, including sedation, constipation, dependence, and, in some cases, opioid-induced hyperalgesia. A critical tension therefore exists between adequate analgesia and risk mitigation. While guidelines from organizations such as the American Society of Clinical Oncology emphasize appropriate opioid access for cancer pain, real-world practice often reflects a more conservative approach, shaped by stigma, regulatory pressure, and clinician uncertainty.

### 8.2. Racial and Socioeconomic Disparities in Pain Management

Substantial evidence demonstrates persistent disparities in cancer pain treatment across racial and socioeconomic groups. Multiple studies have shown that patients from racial and ethnic minority backgrounds are less likely to receive adequate analgesia, timely opioid prescriptions, or referrals to specialist palliative care services compared to White patients. While some data attribute these differences to variations in access to care, insurance coverage, and healthcare infrastructure, other studies highlight the role of implicit bias and structural inequities in clinical decision-making. Importantly, conflicting findings exist: certain large-scale registry analyses suggest that when standardized protocols are implemented, disparities in opioid prescribing may narrow, indicating that system-level interventions can partially mitigate inequities. Nevertheless, the overall body of evidence supports the conclusion that disparities in pain management are multifactorial, involving patient-level, provider-level, and systemic determinants. These inequities contribute not only to worse pain control but also to reduced quality of life and patient-reported outcomes in underserved populations.

### 8.3. Cannabinoids and Inconsistent Clinical Evidence

Cannabinoid-based therapies represent one of the most debated areas in contemporary cancer pain management. Preclinical studies and patient-reported outcomes suggest potential analgesic, antiemetic, and appetite-stimulating effects mediated through the endocannabinoid system. However, clinical trial data remain inconsistent. Some randomized controlled trials and meta-analyses report modest reductions in cancer-related pain with cannabinoid use, particularly as adjunctive therapy to opioids. Conversely, other well-designed studies demonstrate minimal or no statistically significant benefit compared with placebo, especially in objective pain score endpoints. This heterogeneity in findings may be attributed to differences in cannabinoid formulations, dosing regimens, study populations, and outcome measures. Furthermore, concerns persist regarding adverse effects such as dizziness, cognitive impairment, sedation, and potential drug–drug interactions. As a result, major oncology guidelines, including those from the European Society for Medical Oncology, currently regard cannabinoids as adjunctive or experimental options rather than standard-of-care analgesics. Overall, while cannabinoids remain an active area of research, their clinical role in cancer pain management is still evolving, and robust, large-scale, standardized trials are required to clarify their efficacy and safety profile.

## 9. Emerging Therapies and Future Directions in the Field

Several promising developments in oncology and pain management research aim to enhance analgesic effectiveness, reduce the risk of opioid-related complications, and offer new hope for patients with treatment-resistant cancer pain. These future directions have the potential to significantly improve cancer pain management and patient quality of life, driven by advancements in personalized medicine, innovative pharmacological targets, and integrated models.

### 9.1. Cannabinoids

Cannabinoid-based therapies, particularly those involving tetrahydrocannabinol (THC), have garnered increasing interest in their role in cancer-related pain management. A growing proportion of patients are using cannabis and cannabinoid-based therapies to address both palliative and non-palliative cancer-related pain, as well as other associated symptoms [77]. The endogenous cannabinoid system (ECS), which regulates various physiological processes, including pain perception, inflammatory responses, appetite control, energy regulation, cognitive functions like memory and attention, and reward pathways, is the primary mechanism through which THC produces its effects [78]. Currently, two cannabinoid receptor agonists, THC and nabilone, are approved for clinical use in managing chemotherapy-induced nausea and vomiting in cancer patients, and for increasing appetite in individuals with AIDS-related cachexia [78]. Current clinical guidelines from the European Society for Medical Oncology position cannabinoids as adjunctive or experimental rather than first-line analgesics. Overall, while cannabinoids may offer benefit in select patients, their efficacy remains insufficiently established for routine oncologic pain management. Clinical trials that rigorously assess long-term efficacy, optimal dosing, and safety profiles are essential for facilitating wider acceptance in oncology practice.

### 9.2. Gene-Targeted Therapies

Traditional pharmacological agents often lack anatomical specificity and can cause off-target effects, sometimes creating new sources of discomfort for patients [79]. Therefore, pharmacogenomics is increasingly recognized as a relevant component of precision medicine in cancer pain management, particularly in optimizing opioid selection, dosing, and safety. Consistent with the broader movement toward individualized supportive oncology care described in American Society of Clinical Oncology and European Society for Medical Oncology guidance on supportive care and symptom management, genetic variability in drug metabolism, especially involving CYP2D6, has important clinical implications for opioid therapy. Advancements in genomics and molecular profiling are anticipated to allow clinicians to predict opioid responsiveness, individual pain susceptibility, and risks of adverse events, thereby creating a personalized analgesic regimen [80]. Various gene delivery systems have been investigated in preclinical models for targeting pain pathways. Gene delivery to sensory neurons implicated in pain transmission has shown potential using viral vectors such as adenovirus (Ad), adeno-associated virus (AAV), lentivirus, and herpes simplex virus (HSV) [79]. Non-viral methods are also being explored, including lipid nanoparticles, polymer carriers, and other nanotechnologies. Each platform offers distinct advantages and challenges in terms of safety, tissue-targeted delivery, immunogenicity, and clinical feasibility [81]. Concurrently, advances in gene-based research have enabled a more individualized approach to pain management by investigating SNPs linked to the metabolic processing of opioids (e.g., CYP2D6), pain sensitivity (e.g., COMT), and inflammatory responses.

Opioids such as codeine and tramadol are prodrugs that require CYP2D6-mediated conversion into active metabolites. Interindividual differences in CYP2D6 activity result in four clinically relevant metabolizer phenotypes: poor, intermediate, normal (extensive), and ultra-rapid metabolizers. Poor metabolizers exhibit minimal or absent CYP2D6 function, resulting in reduced formation of active metabolites and consequently suboptimal analgesic response. In accordance with guideline-based opioid stewardship principles, these patients are better managed with opioids that do not rely on CYP2D6 metabolism, such as morphine, hydromorphone, or fentanyl, rather than dose escalation of prodrugs. Intermediate metabolizers demonstrate reduced enzymatic activity and may experience attenuated analgesia. Clinical guidelines emphasize the need for individualized titration and close monitoring for inadequate pain control, with consideration of alternative opioids if response remains insufficient. Normal (extensive) metabolizers exhibit expected enzymatic function and generally achieve predictable analgesic outcomes with standard dosing strategies. Ultra-rapid metabolizers have increased CYP2D6 activity, leading to rapid and excessive conversion of prodrugs into active opioid metabolites. Both ASCO and ESMO-aligned safety principles highlight the associated risk of opioid toxicity, including sedation and respiratory depression, even at standard doses. In such patients, avoidance of CYP2D6-dependent opioids is recommended, with preference for non-CYP2D6-dependent agents.

In line with contemporary guideline-based practice, pharmacogenomic testing may be particularly useful in patients with unexpected opioid toxicity, inadequate analgesia, or a history suggestive of atypical opioid response. However, both ASCO and ESMO emphasize that routine preemptive genetic testing is not yet universally recommended, due to limited prospective evidence and cost-effectiveness considerations. Emerging data also suggest that additional genetic factors, including COMT polymorphisms, may influence pain perception and opioid requirements, although these biomarkers are not yet part of standard clinical implementation [82].

Overall, pharmacogenomic-informed opioid selection represents a key component of evolving precision supportive oncology. While not yet fully integrated into routine guideline-mandated practice, it aligns with ASCO and ESMO priorities of improving safety, individualizing analgesic therapy, and reducing preventable opioid-related harm. This expanding study area allows for the development of personalized analgesic regimens tailored to a patient’s genetic makeup. In the future, identifying such genetic profiles may help clinicians predict treatment response, minimize adverse effects, and optimize pain control strategies [81].

### 9.3. Novel Non-Opioid Analgesics and Molecular Targets

Several novel non-opioid analgesics targeting alternative molecular pathways, including TRP channels, neuroimmune interactions, and sodium channels, are currently in preclinical and early clinical development [83]. Early research efforts primarily centered on TRP channels found in pain-sensing neurons. Several potent small-molecule inhibitors targeting TRPV1, TRPV3, and TRPA1 have already advanced into clinical trials as potential new analgesics. More recently, there has been a notable increase in research expanding TRP channel-targeted therapies to additional conditions, including cancer, asthma, metabolic disorders, and many more. Gaining deeper insights into the roles of TRP channels in both normal physiology and disease states may ultimately enable the development of groundbreaking drugs for these challenging medical conditions [84]. Despite these promising findings, clinical translation has been challenging. Several compounds that showed efficacy in preclinical studies failed to demonstrate meaningful benefit in human trials due to issues such as off-target effects, limited therapeutic windows, and safety concerns. As a result, most TRP-channel and neuroimmune-targeted therapies remain in early-phase clinical development.

### 9.4. Critical Appraisal and Translational Limitations

Across all emerging modalities, a consistent theme is the gap between preclinical efficacy and clinical effectiveness. Key limitations include small sample sizes, short trial durations, heterogeneity in pain measurement tools, and inconsistent endpoint definitions. These factors make it difficult to compare studies or establish standardized treatment protocols. Additionally, many emerging therapies face practical barriers related to cost, regulatory approval, and scalability in routine oncology care. As a result, while these interventions represent important future directions, their current role in cancer pain management remains largely investigational.

### 9.5. Integrative and Technological Approaches to Cancer Pain Care

Future models of cancer pain care will increasingly emphasize comprehensive multimodal approaches that integrate rehabilitative, pharmacological, and psychological interventions. The utilization of telemedicine platforms, digital health tools, and wearable biosensors is expected to improve real-time symptom monitoring, enhance patient engagement and adherence, and facilitate individualized treatment adjustments [85,86]. All of these advancements, combined with the early and systematic integration of palliative care, point to a transformative shift toward more precise, equitable, and holistic cancer pain management strategies, with the potential for significantly improved quality of life and patient-centered outcomes.

## 10. Current Research Gaps

Despite significant advances in cancer pain management, several critical research gaps continue to limit the development of fully optimized, evidence-based, and personalized therapeutic strategies. These gaps are particularly evident in comparative effectiveness research, emerging therapies, translational precision medicine, and outcome standardization.

### 10.1. Lack of Head-to-Head Comparisons in Multimodal Pain Management

One of the most important limitations in the current literature is the absence of robust head-to-head clinical trials comparing different multimodal cancer pain regimens. While multimodal analgesia is widely recommended, most available studies evaluate single interventions or additive effects rather than systematically comparing combinations of pharmacologic and non-pharmacologic strategies. As a result, there is insufficient evidence to determine the relative superiority of specific combinations (e.g., opioid + anticonvulsant vs opioid + antidepressant vs opioid + interventional approaches) across different pain phenotypes. This lack of comparative effectiveness data limits the ability to develop standardized, mechanism-based treatment algorithms tailored to neuropathic, visceral, or somatic cancer pain.

### 10.2. Methodological Heterogeneity in Cannabinoid Research

Cannabinoid-based pain research is characterized by significant methodological heterogeneity, which substantially limits evidence synthesis and meta-analytic interpretation. Studies vary widely in terms of cannabinoid composition (THC-dominant, CBD-dominant, or balanced formulations), routes of administration, dosing strategies, treatment duration, and outcome measures. In addition, pain outcomes are often assessed using non-standardized scales or subjective patient-reported endpoints without consistent definitions of clinically meaningful improvement. This variability contributes to inconsistent findings across trials and complicates efforts to establish clear evidence-based recommendations for clinical use.

### 10.3. Translational Barriers in Pharmacogenomic Implementation

Although pharmacogenomic associations such as CYP2D6 and COMT polymorphisms have been well described in relation to opioid metabolism and pain sensitivity, their translation into routine clinical practice remains limited. Key barriers include the absence of large-scale prospective interventional trials demonstrating improved patient outcomes, variability in access to genetic testing, and a lack of standardized clinical decision-support tools. Furthermore, uncertainty remains regarding cost-effectiveness, optimal testing timing, and how best to integrate pharmacogenomic data into real-time prescribing decisions in oncology settings. As a result, pharmacogenomic-guided analgesia remains largely investigational rather than a routine component of cancer pain management.

### 10.4. Lack of Standardized Cancer Pain Assessment Frameworks

A further major limitation is the absence of universally standardized and validated pain assessment tools that capture the multidimensional nature of cancer-related pain. Existing instruments often focus primarily on pain intensity, without adequately capturing neuropathic characteristics, breakthrough pain patterns, emotional distress, or functional impairment. This lack of standardization reduces comparability across studies and clinical settings, limiting the ability to perform high-quality meta-analyses and develop unified treatment guidelines. Additionally, variability in assessment practices across institutions contributes to inconsistent pain recognition and management in routine oncology care.

Collectively, these gaps highlight the need for future research focused on comparative effectiveness trials, standardized outcome measures, and translational studies bridging molecular insights with clinical decision-making. Addressing these limitations is essential to advance cancer pain management toward a more precise, evidence-driven, and patient-centered discipline.

## 11. Conclusions

Cancer-related pain remains one of the most complex and burdensome symptoms experienced by patients, often significantly diminishing quality of life. Malignancies such as lung cancer, breast cancer, and hematologic cancers are frequently associated with substantial pain burden arising from both disease progression and treatment-related complications. These conditions often present with neuropathic, visceral, and somatic pain phenotypes, each requiring mechanism-specific management. While opioids remain a cornerstone of treatment, their limitations, including adverse effects, misuse potential, and societal stigma, underscore the need for broader, individualized strategies. Adjuvant analgesics, topical agents, and non-pharmacological interventions expand the therapeutic landscape, enabling more precise and multimodal approaches to pain control.

Emerging therapies, including cannabinoid-based treatments and gene-targeted strategies, offer promise for more personalized and effective pain management, particularly in refractory cases. Advancing cancer pain management will require continued integration of multidisciplinary care, evidence-based innovation, and patient-centered approaches. Addressing pain as a central component of oncology care is essential to improving both clinical outcomes and quality of life.

## Figures and Tables

**Table 1:** Common cancer types associated with chronic pain and their dominant mechanisms. Pain subtypes vary by cancer and may arise from tumor progression, metastasis, or treatment-related effects. Blank cells indicate that the respective pain subtype is not a dominant presentation for that cancer type based on current literature.

Cancer Type	Dominant Pain Presentations		
Lung Cancer	Somatic Pain	Visceral Pain	Neuropathic Pain
Breast Cancer	Somatic Pain (Bone, Chest Wall)		Neuropathic Pain
Leukemia	Bone Pain	Visceral Compression	Mixed Pain
Lymphoma	Bone Pain	Visceral Compression	

## Data Availability

No new data were created or analyzed in this study. Data sharing is not applicable to this article.

## Abbreviations

CBT	Cognitive Behavioral Therapy
NINPT	Non-Invasive, Non-Pharmacological Therapies
TENS	Transcutaneous Electrical Nerve Stimulation
RCT	Randomized Controlled Trial
WHO	World Health Organization
ASCO	American Society of Clinical Oncology
TCA	Tricyclic Antidepressant
SNRI/SNRIs	Serotonin–Norepinephrine Reuptake Inhibitor(s)
CS	Corticosteroids
GABA	Gamma-Aminobutyric Acid
CGRP	Calcitonin Gene-Related Peptide
TRPV1/TRPV3/TRPA1	Transient Receptor Potential Channels
PLA2	Phospholipase A2
COX-2	Cyclooxygenase-2
CYP2D6	Cytochrome P450 2D6
COMT	Catechol-O-Methyltransferase
SNPs	Single-Nucleotide Polymorphisms
Ad	Adenovirus
AAV	Adeno-Associated Virus
HSV	Herpes Simplex Virus
THC	Tetrahydrocannabinol
